# School-level determinants of incidence of sports-related concussion: Findings from the CARE Consortium

**DOI:** 10.1371/journal.pone.0284259

**Published:** 2023-04-10

**Authors:** Bhavna Singichetti, Stephen W. Marshall, Katherine M. Breedlove, Kenneth L. Cameron, Michael A. McCrea, Thomas W. McAllister, Steven P. Broglio, CARE Consortium Investigators

**Affiliations:** 1 Injury Prevention Research Center, University of North Carolina at Chapel Hill, Chapel Hill, NC, United States of America; 2 Department of Epidemiology, Gillings School of Global Public Health, University of North Carolina at Chapel Hill, Chapel Hill, NC, United States of America; 3 Department of Radiology, Center for Clinical Spectroscopy, Brigham and Women’s Hospital, Harvard Medical School, Boston, MA, United States of America; 4 John A. Feagin Jr. Sports Medicine Fellowship, Keller Army Hospital, United States Military Academy, West Point, NY, United States of America; 5 Department of Neurosurgery, Medical College of Wisconsin, Milwaukee, WI, United States of America; 6 Department of Psychiatry, Indiana University School of Medicine, Indianapolis, IN, United States of America; 7 Michigan Concussion Center, University of Michigan, Ann Arbor, MI, United States of America; 8 School of Kinesiology, University of Michigan, Ann Arbor, MI, United States of America; University of Virginia, UNITED STATES

## Abstract

**Objective:**

Epidemiologic research on sports-related concussion (SRC) has focused on individual risk factors, with limited research on institutional risk factors and variability in concussion rates.

**Methods:**

This study used data from 53,822 athletes-seasons collected at 30 United States sites (26 civilian institutions and 4 military service academies), from 2014/15 to 2018/19 academic years, by the Concussion Assessment, Research, and Education Consortium. School-level risk factors included competitive division (DI, DII, DIII), school type (military/civilian) and a Sport Risk Index (SRI; Low, Medium, High). For comparability between civilian institutions and military academies, only NCAA athletes and concussions in sports games and practices were included. Random intercepts log-binomial regression was used to estimate Risk Ratios (RRs) and model variability in SRC risk.

**Results:**

A total of 2,503 SRCs were observed during the study period, including 829 competition SRCs (33%) and 1,674 practice SRCs (67%). Most variability in SRC risk was at the level of athlete or team (within-school), rather than at the school-level. Specifically, across the three SRC outcomes (all [competition and practice combined], competition-only, and practice-only), within-school variability was 5 to 7 times greater than between-school variability. Three school-level risk factors (Division, School Type, and SRI) accounted for over one-third (36%) of between-school variability. SRI was the strongest school-level predictor of SRC risk (RR = 5.7; 95%CI: 4.2, 7.6 for High vs. Low). SRC risk was higher for Division I compared to Divisions II/III (RR = 1.6; 95%CI: 0.9, 2.9 for DI vs. DIII), and military academies had a moderately elevated risk of SRC (RR = 1.4; 95%CI: 0.7, 2.7).

**Conclusion:**

A large portion of the apparent variability between schools was attributable to structural factors (sport risk and competitive level), suggesting that there were minimal systemic differences in concussion identification between schools. While most variability is within-school, understanding school-level determinants of concussion risk may still be important in providing the implementation science context for individual-level interventions.

## Introduction

Sports-related concussion (SRC) is a serious public health problem [1]. Numerous prior studies have presented incidence estimates for concussion in various sports and settings [2, 3]. Most research to date on risk factors for SRC has focused on athlete-level risk factors [4]. Little attention has been focused on school-level determinants of SRC risk among collegiate student athletes, and the factors that generate variability in observed SRC risk between schools. Understanding variability in concussion rates, and structural determinants of concussion at the level of the institution, has the potential to inform new areas for prevention efforts and deepen our understanding of factors that influence concussion risk.

Differences in observed concussion incidence among college-aged individuals (college/university and service academy student-athletes) between sports and settings likely reflect interplay between school-level, athlete-level, and team-level factors. School-level factors influence between-school variability, whereas athlete-level and team-level factors influence within-school variability. School-level factors include differences in the mix of sports between schools, experience and expertise of sports medicine staff in concussion identification, intensity of the institutional strategies encouraging concussion identification and reporting, and level of competition (i.e., National Collegiate Athletic Association Division [DI, DII, DIII]). Important athlete-level and team-level risk factors may include frequency and magnitude of head impacts, anatomic and metabolic factors influencing propensity to injury, and perceived team and coach attitudes to concussion prevention and disclosure [5, 6].

In addition to school-level factors noted above, methodologic complexities in ascertainment and reporting of concussion [7, 8], and nuances in the determination of athletes at risk and the computation of incidence measures [9] are other potential sources of between-school variation in observed concussion risk. Understanding the role of methodologic factors is important because it has potential to suggest improvements in research methodology in future studies.

This study aimed to advance understanding of the role of school-level factors using a statistical model for variability in SRC risk. Specifically, the purpose of this study was to 1) model between-school and within-school variability in observed SRC incidence in schools participating in a multi-site study and 2) identify school-level determinants of variability in the reported SRC incidence. We hypothesized that we would observe significant variability both between school and within school, and that these two components of overall variability would be similar in magnitude.

## Methods

We used data from the Concussion Assessment, Research and Education (CARE) Consortium, established by the National Collegiate Athletic Association (NCAA) and the US Department of Defense (DOD) in an effort to better understand the clinical effects of SRC across multiple sports and institutions in both men and women. CARE is the largest prospective study of SRC conducted to date [10].

The CARE Consortium organizational structure and methods have been detailed elsewhere [10]. The CARE Consortium quantified SRC incidence and provided a longitudinal natural history of SRC in a large sample of collegiate athletes [10]. CARE is a multisite study of 30 collegiate institutions (26 civilian institutions and 4 military service academies) that enrolled over 41,000 military cadets and student-athletes (about 90% of all eligible athletes) and registered over 2,500 SRCs. Institutional Review Board approval was obtained locally for each site as well as through the Department of Defense Human Research Protection Office. Written informed consent was obtained for participants 18 years and older, and assent with parental/guardian consent was obtained for participants younger than 18 years old. This study was conducted in accordance with the Declaration of Helsinki.

### Data collection and study participants

Data from the 2014/15 to 2018/19 academic years were included in these analyses. Annual pre-season/baseline assessments were administered to all consenting athletes and those sustaining an identified concussion, with up to five standardized post-injury evaluations completed up to 6 months following injury. We used a risk-orientated framework [11], so multiple concussions to the same athlete, in same team, in the same season and at the same school, were counted only once in this analysis. Removing this assumption (i.e., using all concussions) had no effect on the analyses. All schools in the CARE Consortium used a consistent definition for concussion [12] and used standardized entry criteria for enrolling athletes into the study [10].

The analysis in this paper excluded concussions occurring outside of the sporting environment (e.g., motor vehicle crash). We also excluded military cadets who were not NCAA athletes in a nationally-sanctioned championship collegiate sport, i.e., this analysis was limited to NCAA student-athletes at all schools, military and civilian. These restrictions were imposed in order to create an etiologically homogeneous set of data for analysis.

CARE did not collect competition exposure data in terms of games or minutes played, nor did it collect training exposure data. Therefore, we used athlete-seasons as the denominator for time at risk for the athletes [9]. One athlete-season at risk was considered to be one athlete participating for one season on a single team. For example, an athlete who was in the study for three years on the basketball team contributed three athlete-seasons at risk [9]. For civilian sites, the number of athletes on each team in each season was based on the number of athletes baseline tested by CARE investigators at each site. For military sites, this was estimated based on institutional attrition from the academy program.

### School-level risk factors

The school-level determinants of SRC incidence examined in this study included competitive division (NCAA DI, DII, DIII) and school type (military or civilian). Additionally, a 3-level Sport Risk Index (SRI; Low, Medium, High) was assigned for each sport (Table 1). The SRI ratings were intended as working classification of sports into three categories based on measures of SRC risk. We began with a previously published expert panel 3-level classification of contact in youth sports [13]. In order to make the 3-level classification specific to the CARE study population, we updated it using the most recent empirical data on the incidence of concussion in US collegiate sports [2], following by expert review and the independent review by two of the authors (KMB, SWM). The resulting measure provides a respectable proxy for SRC risk at the level of a sport. Our scientific interest in this analysis, however, was school (not sport) level SRC incidence. To examine SRI as a school-level determinant of SRC risk, we created a sport-school-year database that provided a count of the total number of consented athletes, and the subset with one or more registered SRC(s), in each season, in each sport, for each CARE school. This allowed us to model school-level SRI as the “mix” of high, medium, and low risk sports for each school.

**Table 1:** Concussion risk categories and injury frequencies by sport, CARE CSC, 2014/15 to 2018/19 academic years.

Sport	Risk Category	Seasons		Sports Concussions			Sport	Risk Category		Seasons			Sports Concussions		
		Team	Player	All	Competition	Practice				Team		Player	All	Competition	Practice
*Sports Played By Both Sexes*															
***Female***							***Male***								
**Basketball**	High	118	1579	124	35	89	**Basketball**		Medium		112	1977	95	25	70
**Cheerleading**	Low	49	1172	57	4	53	**Cheerleading**		Low		36	380	12	2	10
**Cross Country/Track**	Low	116	3516	23	9	14	**Cross Country/Track**		Low		108	3024	11	1	10
**Diving**	Low	70	291	30	2	28	**Diving**		Low		60	210	18	1	17
**Fencing**	Low	18	218	3	1	2	**Fencing**		Low		15	185	2	2	0
**Field Event**	Low	95	809	16	5	11	**Field Event**		Low		86	679	5	1	4
**Golf**	Low	59	541	1	1	0	**Golf**		Low		69	1044	2	0	2
**Gymnastics**	Medium	51	780	49	4	45	**Gymnastics**		Medium		19	323	19	3	16
**Ice Hockey**	High	9	195	15	8	7	**Ice Hockey**		High		26	530	53	44	9
**Lacrosse**	Med	53	1368	66	22	44	**Lacrosse**		Med		35	1345	90	26	64
**Soccer**	High	113	3039	207	110	97	**Soccer**		High		96	2876	152	86	66
**Swimming**	Low	87	1971	49	4	45	**Swimming**		Low		72	1763	9	0	9
**Tennis**	Low	99	853	15	4	11	**Tennis**		Low		91	921	8	3	5
**Volleyball**	Medium	108	1704	132	34	98	**Volleyball**		Medium		20	260	11	7	4
**Water Polo**	Medium	25	504	42	8	34	**Water Polo**		Medium		17	398	26	7	19
*Sports Played By One Sex*															
***Female***							***Male***								
**Field Hockey**	Medium	95	809	16	5	11	**Baseball**		Low		106	3682	44	22	22
**Softball**	Medium	104	1984	52	18	34	**Football**		High		118	11131	931	286	645
							**Wrestling**		High		63	1644	99	35	64
*Mixed Sports (Female & Male on the Same Team)*															
**Rifle**	Low	32	369	2	0	2	**Other**		Low		20	166	4	2	2
**Rowing/Crew**	Low	65	3461	13	2	11									

### Statistical analysis

To model variability in SRC risk at the school level, we used a generalized linear mixed model [14] and included a random intercept probability distribution for the effect of school. Thus, the model partitioned overall variability in SRC risk into between-school variability and within-school variability. Three concussion outcomes were modeled: the risk of any SRC (competition and practice SRCs combined), the risk of competition SRC, and the risk of practice SRCs only. Because all three outcomes were SRC risks, we used a log link model with binomial probability distribution for residuals [15]. The denominator (“offset”) used in these models was the number of student-athletes consenting to participate in CARE at each school in each year of the study. The general form of the model was:

$$E[{SRC}_{ij}]=\beta_{0j}+\beta_{1}{\mathrm{RiskIndex}}_{ij}+\beta_{2}{\mathrm{Division}}_{j}+\beta_{3}{\mathrm{SchoolType}}_{j}+n_{ij}$$

where *SRC*_*ij*_ was the SRC count for the *i*^th^ team at the *j*^th^ school, RiskIndex_*ij*_ was the SRI for each sport (Table 1), Division_*j*_ was the school’s competitive league, SchoolType_*j*_ was a binary variable coding military or civilian school, and *n*_*ij*_ was the number of athletes on the *i*^th^ team at the *j*^th^ school.

We used SAS’s PROC GLIMIX (Cary, NC) G-side estimation methods to include the random intercepts for school [16]. We used robust (empirical) sandwich estimates to estimate the fixed effect standard errors, incorporating Morel-Bokossa-Neerchal small-sample bias correction to account for the fact that only 30 schools (clusters) were available [17]. The model was fit using a maximum likelihood-based method, adaptive Gaussian quadrature, in order to obtain unbiased variance estimates for the random effects [16].

To identify school-level risk factors for SRC, we computed risk ratios (RRs), with corresponding 95% confidence intervals (CIs) and p-values, for each variable in a series of univariate models (Table 2). This model was also used to partition the overall variance of each SRC outcome (all SRCs, competition-only SRCs, and practice-only SRCs) as shown in Table 3. We also fit a multivariate model for each SRC outcome to obtain fully adjusted RRs (Table 2) and obtain an estimate within-school variability, adjusted for school-level predictors (Table 3).

**Table 2:** Estimated variance^1^ in sports-related concussion, CARE CSC, 2014/15 to 2018/19 academic years.

	School-Level Risk Factors Included in Model (% Change from Null Model)				
	Univariate Models				Multivariate Model
	No School-Level Factors in model	Sport Risk Index in Model	Division in Model	School Type in Model	All 3 Factors (Risk Index, Division, Type)
*All Sports-Related Concussions (Competition and Practice Combined)*					
**Within-School**	2.14 (ref.)	2.17 (+1%)	2.14 (0%)	2.14 (0%)	2.17 (+1%)
**Between-School**	0.28 (ref.)	0.24 (-14%)	0.24 (-14%)	0.27 (-4%)	0.18 (-36%)
*Competition Concussions Only*					
**Within-School**	1.47 (ref.)	1.45 (-1%)	1.47 (0%)	1.47 (0%)	1.44 (-2%)
**Between-School**	0.27 (ref.)	0.21 (-22%)	0.27 (0%)	0.25 (-7%)	0.18 (-33%)
*Practice Concussions Only*					
**Within-School**	1.87 (ref.)	2.13 (+14%)	1.87 (0%)	1.87 (0%)	2.14 (+14%)
**Between-School**	0.33 (ref.)	0.30 (-9%)	0.24 (-27%)	0.31 (-6%)	0.19 (-42%)

^1^Variance estimates from log-binomial regression model, includes random intercepts for school

**Table 3:** School-level risk factors for sports-related concussion, CARE CSC, 2014/15 to 2018/19 academic years.

	Univariate				Multivariate			
	Risk Ratio (95% CI)		P-value relative to ref.	Overall P-value	Risk Ratio (95% CI)		P-value relative to ref.	Overall P-value
***All Sports-Related Concussions (Competition and Practice Combined)***								
**Sport Risk Index**								
Low	1	(reference)			1	(reference)		
Medium	4.17	(3.35, 5.20)	< .0001	< .0001	4.18	(3.33, 5.25)	< .0001	< .0001
High	5.66	(4.25, 7.53)	< .0001		5.66	(4.23, 7.59)	< .0001	
**Division**								
D1	1.52	(0.90,2.59)	0.1139	0.1734	1.63	(0.90, 2.94)	0.1040	0.1497
D2	0.99	(0.39, 2.47)	0.9771		1.05	(0.42, 2.61)	0.9097	
D3	1	(reference)			1	(reference)		
**School Type**								
Civilian	1	(reference)			1	(reference)		
Military	1.47	(0.58, 3.72)	0.4047	0.3975	1.41	(0.73, 2.73)	0.2983	0.2886
***Competition Concussions Only***								
**Sport Risk Index**								
Low	1	(reference)			1	(reference)		
Medium	5.60	(4.03, 7.78)	< .0001	< .0001	5.60	(3.94, 7.96)	< .0001	< .0001
High	10.61	(8.07, 13.96)	< .0001		10.63	(7.91, 14.28)	< .0001	
**Division**								
D1	1.15	(0.51, 2.61)	0.7205	0.8312	1.24	(0.57, 2.73)	0.5706	0.7099
D2	0.96	(0.35, 2.67)	0.9428		1.03	(0.40, 2.67)	0.9433	
D3	1	(reference)			1	(reference)		
**School Type**								
Civilian	1	(reference)			1	(reference)		
Military	1.61	(0.81, 3.20)	0.1637	0.1526	1.53	(0.88, 2.66)	0.1225	0.1104
***Practice Concussions Only***								
**Sport Risk Index**								
Low	1	(reference)			1	(reference)		
Medium	3.83	(3.05, 4.82)	< .0001	< .0001	3.83	(3.01, 4.88)	< .0001	< .0001
High	4.41	(3.17, 6.13)	< .0001		4.42	(3.16, 6.18)	< .0001	
**Division**								
D1	1.99	(1.27, 3.11)	0.0041	0.0049	2.09	(1.16, 3.77)	0.0165	0.0215
D2	1.11	(0.43, 2.88)	0.8177		1.18	(0.43, 3.20)	0.7374	
D3	1	(reference)			1	(reference)		
**School Type**								
Civilian	1	(reference)			1	(reference)		
Military	1.44	(0.49, 4.20)	0.4952	0.4895	1.39	(0.70, 2.79)	0.3335	0.3244

## Results

This analysis included a total of 53,822 athlete-seasons in 37 collegiate championship sports at 30 CARE schools (4 military academies and 26 civilian institutions). Athletes-seasons from both male (n = 32,4009, 60%) and female (n = 21,413, 40%) student-athletes were included (Table 1). A total of 2,503 SRCs were observed, including 829 competition-related (33%) and 1,674 practice-related (67%) SRCs. The overall risk of SRC was 46.5 per 1,000 athlete-seasons.

### Magnitude and determinants of school-level variability

Most of the observed variability in concussion risk was at the individual and team level, rather than at the school level (Table 2). For all SRC, competition-only SRC, and practice-only SRC risk, respectively, variability at the level of the team and individual (within-school) was between 7.6, 5.4, and 7.5 times greater than the variability at the level of the schools (between-school) in unadjusted models (Table 2). After adjustment for school-level factors, within-school variability was between 12.1, 8.0, and 11.3 times greater than the between-school variability, for all SRC, competition-only SRC, and practice-only SRC risk, respectively.

In combination, the three school-level risk factors (Division, military vs. civilian school, and SRI) accounted for 36% of the variation between schools for all SRCs, 33% for competition SRCs, and 42% for practice SRCs. For all SRC and practice-only SRCs, SRI and Division were the most important determinants of school-level variability. For competition SRCs, only SRI was an important determinant (Table 2). Adjustment for school-level risk factors had minimal impact on the estimated variance at the individual and team level.

### School-level risk factors for SRC

In both univariate and fully adjusted models across all three concussion outcomes, SRI was the strongest predictor of SRC risk. The risk of concussion increased with each increment of SRI level (Table 3). For all SRC, the largest step-up in the estimated risk involved moving from Low to Medium (multivariate-adjusted Risk Ratio [RR] = 4.18; 95%CI: 3.33, 5.25). Moving from Medium to High further increased the estimated risk, but the increment in estimated risk was much smaller (multivariate-adjusted High versus Low RR = 5.66; 95%CI: 4.23, 7.59), thus RRs comparing High to Low were only moderately greater than those comparing Medium to Low.

In all models, overall concussion risk tended to be higher in Division I schools than Division III schools (multivariate-adjusted RR = 1.63; 95%CI: 0.90, 2.94). Overall concussion risk was very similar for Division II schools and Division III schools, in both univariate and multivariate models. These patterns were also true for the models limited to competition-only and practice-only SRC risk (Table 3).

As mentioned above, all analyses in this paper were limited to collegiate athletes only. Therefore, at the military academies, only military cadets who were collegiate athletes were included in our models (i.e. we excluded military cadets who were not NCAA athletes). Even with this restriction, military academies tended to have an elevated risk of SRC relative to civilian institutions. This was true for overall SRC risk (multivariate-adjusted RR = 1.41; 95%CI: 0.73, 2.73), and for competition-only (multivariate-adjusted RR = 1.53; 95%CI: 0.88, 2.66) and practice-only SRC risk (multivariate-adjusted RR = 1.39; 95%CI: 0.70, 2.79).

## Discussion

While numerous studies have reported estimates of SRC incidence, none have explored the determinants of variability in concussion incidence between schools. Given that concussion has a complicated and heterogeneous clinical presentation and subject to management by individual clinical providers, our *a priori* expectation was that there would be substantial variability between schools participating in a multisite study of concussion. Contrary to our hypothesis, there was far more variability at the individual and team level (within-school) than at the school level (between-school). Specifically, across the three outcomes (all SRCs, competition-only SRCs, and practice-only SRCs), the within-school variance was 5 to 7 times greater than the between-school variance in unadjusted models. This suggests that any systemic differences in concussion identification between the institutions participating in the CARE Consortium were minimal, relative to variability within the same institution. The use of CARE standard operating procedures, in addition to the use of standard clinical NCAA concussion management guidelines [18], may have heightened standardization between schools and decreased between-school variability.

Not only did school-level variability account for only a small portion of the overall variability, it was largely attributable to known factors. Most of the school-level variability was due to two readily-measured school-level factors (Division and SRI). Division was a particularly important determinant of variance in practice, which may be due to the natural variation in practice drills, practice facilities, and practice frequency between division. SRI may be a more important determinant of variance in competition because concussion rates are higher in competition than in practice, and the competition-to-practice rate ratio varies considerably by sport.

School-level factors can create uncontrolled variability if they are not accounted for in an analysis. Therefore, measuring and accounting for school-level factors may improve precision and power in future studies. However, it must be noted that accounting for school-level factors does not remove individual and team variability, which is the major source of variability in SRC risk. Finally, like Division and School Type, which differs from school to school, the SRI used in this analysis reflects the composite measure at an institution based on the mix of high, medium and low risk sports at each school. However, Division and School are discrete fixed factors, whereas there are many possible combinations of the NCAA sports offered at a collegiate institution [19]. The large number of possible combinations, and the resulting variability between schools in the mix of high, medium, and low risk sports offered, likely resulted in SRI emerging as an important determinant of school-level SRC risk.

This study provides initial information that advances our understanding of determinants of concussion incidence variability between schools. It also suggests strategies and procedures for measuring and mitigating between-site variability in future multisite studies, such as centralized adjudication of potential SRC cases by the coordinating center. Clearly, standard operating procedures for identification of SRCs [18] is an important aspect of any large multisite study of SRC. However, these design considerations need to be balanced against equity considerations, including representation of sites with less clinical and research resources in large prospective studies. For example, limiting a large study to just one division would reduce variability but would compromise representativeness and generalizability. Therefore, it is important to consider statistical approaches that allow adjustment for variability, particularly since naïve analyses that fail to account for school-level determinants of variability can create a false impression of high and unexplainable variability between sites.

This study was limited in terms of the school-level factors that could be examined. The schools in this study used standardized procedures for enrollment and concussion identification [10]. Thus, it may not be appropriate to generalize beyond this sample to the broader population of US universities. The study was also limited to just three school-level factors. Other school-level factors may create variability in concussion risks could include variability in practice regimes and concussion education, and the role of peer, coach, and institutional influences [20]. These factors were not measured in CARE. An additional limitation associated with CARE is the lack of exposure data that is more finely detailed than athlete-seasons. Unfortunately, CARE did not track individual-level or team-level participation in competitions and practices. Differences between Divisions, and between military academies and civilian institutions, are not accounted for in our athlete-seasons-based analysis. In order to maximize comparability between the military and civilian sites, we limited the analyses in this paper to sports-related concussions.

We observed that the military sites had a moderately elevated risk relative to the civilian schools for all three SRC outcomes. There are several potential factors that could account for this apparent increased risk at the military sites. These could include differences between military and civilian athletes in individual-level factors that we did not measure, such as increased subclinical head impact exposure outside of sports through military training, or the perceived importance of disclosing concussion symptoms [6, 20–22]. Differences in institutional policies may also play a role, and these may reflect variations between schools in the implementation of concussion identification programs, which may be implemented at higher level of intensity at some of the military academies, relative to the average of the civilian sites. Finally, participants at military academies may have behavioral risk factors for sports-related concussion, such as risk-taking, that may be more prevalent in the military academy setting [21, 22]. These factors may contribute to the increased risk observed in the current study.

In conclusion, we found that most of the observed variability in concussion risk was at the individual and team level, rather than at the school level. A large portion of the variability between schools in SRC risk in CARE was due to two readily-measured factors, sport risk and level of competition (division). Within-school variation was considerably larger than between-school variation, suggesting that there were few, if any systematic differences between schools in SRC identification. Additionally, while the results from this paper clearly indicate that individual-level factors account for the majority of variance in SRC incidence, school-level factors should not be ignored, since institutional support is likely key to implementation and adoption of SRC interventions, such as concussion awareness programs. Understanding school-level determinants and variability in concussion risk may help identify school-level opportunities for SRC interventions. These results also emphasize the importance of efforts to standardize clinical and institutional procedures in the design of future multi-site studies.
